# Comparison of individual, group and environmental sampling strategies to conduct influenza surveillance in pigs

**DOI:** 10.1186/s12917-019-1805-0

**Published:** 2019-02-14

**Authors:** Jorge Garrido-Mantilla, Julio Alvarez, Marie Culhane, Jayaveeramuthu Nirmala, Jean Paul Cano, Montserrat Torremorell

**Affiliations:** 1 Veterinary Population Medicine Department, College of Veterinary Medicine, University of Minnesota, St. Paul, MN USA; 2 Centro de Vigilancia Sanitaria Veterinaria (VISAVET), Universidad Complutense, Madrid, Spain; 3 Departamento de Sanidad Animal, Facultad de Veterinaria, Universidad Complutense, Madrid, Spain; 4 PIC, Hendersonville, TN USA

**Keywords:** Environment, Influenza, Pigs, Sampling, Surveillance, Udder wipe

## Abstract

**Background:**

Influenza A virus (IAV) is an important pathogen in pigs that affects productivity and has important public health implications because of its zoonotic nature. Surveillance is central to the control of influenza, however, detection of IAV infections can be challenging in endemically infected herds with low prevalence of infection.

**Methods:**

In groups of suckling (18–21 days of age) and growing (35–45 days of age) pigs, we compared various sampling approaches to detect, isolate and sequence IAV using individual (nasal swabs, nasal wipes and oropharyngeal swabs), group (oral fluids, surface wipes and sow udder skin wipes) and environmental (airborne particles deposited on surfaces and air samples) sampling approaches. All samples were tested by IAV rRT-PCR and a subset was used for virus isolation and direct sequencing.

**Results:**

In general, environmental and group samples resulted in higher odd ratios (range = 3.87–16.5, *p*-value < 0.05) of detecting a positive sample by rRT-PCR compared to individual pooled samples, except for oropharyngeal swabs (OR = 8.07, *p*-value < 0.05). In contrast, individual samples were most likely to yield a viral isolate by cell culture. Oropharyngeal swabs in suckling pigs (78.4%), and nasal swabs (47.6%) or nasal wipes (45%) in growing pigs, and udder wipes in lactating sows (75%) were the preferred samples to obtain an isolate.

**Conclusions:**

Our findings indicate that group and environmental sampling strategies should be considered in influenza surveillance programs in particular if the goal is just to detect infection. This study provides new information on sampling approaches to conduct effective influenza surveillance in pigs and identifies udder wipes from lactating sows as a novel sample type that offers a convenient, cheap and sensitive manner to monitor IAV in litters prior to weaning.

**Electronic supplementary material:**

The online version of this article (10.1186/s12917-019-1805-0) contains supplementary material, which is available to authorized users.

## Background

Influenza A virus (IAV) causes acute respiratory disease in pigs and affects production performance resulting in decreased weight gain and increased feed conversion [1]. Pigs are important in influenza ecology and transmission to other species because they can yield novel reassorted viruses with zoonotic and pandemic potential [2, 3]. Control of IAV in pigs is difficult due to the high genetic diversity of IAV strains, the co-circulation of multiple strains in farms [4] and the on-going introduction of strains of human origin [5]. Although there are strategies to manage the disease (i.e vaccination), reduce clinical signs or prevent secondary infections [6], detecting IAV in endemically infected herds can be challenging, especially in low disease prevalence scenarios where the sample size could be large. Detection and characterization of IAV becomes even more critical in herds undergoing control programs where decisions on pig flow or vaccination strategies are necessary.

Surveillance is central to the control, elimination and prevention of influenza in swine herds. Most surveillance efforts are based on the submission of tissues from clinically affected pigs to diagnostic laboratories (contingent or passive surveillance) or include the regular submission of age appropriate samples, mostly nasal swabs and oral fluids, to monitor the influenza status of herds (routine or active surveillance). In 2010, the United States Department of Agriculture (USDA) and Animal and Plant Health Inspection Service (APHIS) initiated a voluntary influenza surveillance program in order to identify IAVs that circulate in swine, and to acquire IAV strains that could be used to improve animal health diagnostics and vaccines [7]. After 6 years, there was reduced funding for the program, resulting in a drop in sample submissions. Surveillance efforts are expensive particularly if the goal is to obtain a virus isolate and a viral gene sequence. Therefore, influenza surveillance strategies should be a balance between cost, test sensitivity, easinest of sample collection, and the ability to isolate and sequence the virus.

Monitoring diseases in populations using individual samples represents a major limitation because the number of samples required to detect infection can be high and costly [8]. The standard sample type to test for IAV has been nasal swabs, and in recent years oral fluids have become the sample of choice to sample groups of weaned pigs. However, obtaining a viral isolate or a sequence from oral fluids is challenging and obtaining oral fluids from pigs prior to wean is difficult because of the pig’s lack of interest in chewing the rope. Nasal wipes have also been recommended for IAV, in particular to monitor the virus in agricultural fairs [9]. The collection of air or surface samples has also been used in studies to assess the risk of influenza exposure in pig workers [10] or other pigs [11]. In this study we included the collection of udder wipes as a novel sample type to assess the status of litters prior to wean which represents the deposition of nasal and oral secretions of suckling pigs on the udder skin of the sow. To the authors’ knowledge, there has not been a comprehensive side-by-side comparison of sample types to detect, isolate and sequence influenza in pigs prior to and after weaning, which could be useful to design better strategies for IAV surveillance and control in pigs.

In this study, we compared the detection rates of various sample types collected from pigs prior to weaning and after weaning to detect IAV at the individual, group (crate/pen) or environmental (room) level. We evaluated which sampling approach was the best to detect, isolate and sequence IAV directly from the sample. We hypothesize that group and environmental sampling strategies offer higher sensitivity than individual sampling approaches to detect, isolate and sequence influenza viruses.

## Results

### Influenza rRT-PCR

IAV was detected by rRT-PCR in 4 out of 6 breeding herds and 5 out of 6 wean-to-finish farms (Table 1). In positive breeding herds, 78% (31/40, 95% confidence interval 61–89%), 55% (22/40, 95% CI 38–70%) and 53% (21/40, 95% CI 36–68%) of pools of the oropharyngeal swabs, nasal swabs and nasal wipes collected tested rRT-PCR positive, respectively. The Cochran test revealed the presence of differences between the detection rates obtained using different sample types (*p* < 0.05) although the post-hoc tests revealed that these were significant only between nasal wipes and nasal swabs when compared with air/airborne particle deposition samples (*p* = 0.001, *p* = 0.006, *p* = 0.002 and *p* = 0.01 respectively), and between surface wipes and air samples (*p* = 0.03). Detection rates using oropharyngeal swabs were higher than using nasal swabs or nasal wipes although no differences were found (*p* = 0.7 and *p* = 0.4, respectively). No differences were observed when comparing nasal swabs and wipes either (*p* = 1). Regarding group level samples, 78% (31/40, 95% CI 61–89%) and 60% (24/40, 95% CI 43–75%) of the udder and surface crate wipes were positive, respectively. However, differences were not significant (*p* = 1). Of the 40 expected oral fluids from breeding herds only seven could be collected due to the reluctance of the suckling pigs to chew the rope. Out of these, 86% (6/7, 95% CI 42–90%) tested positive. A higher proportion of environmental samples tested positive, with 100% (40/40, 95% CI 91–100%) and 88% (35/40, 95% CI 73–95%) of the air and airborne particle deposition respectively testing positive. No statistical difference was detected between environmental sample types (*p* = 1).

**Table 1:** Detection of influenza A virus by real-time reverse transcription polymerase chain reaction (rRT-PCR) by sample and farm type

Farm type	Farm ID	Farm status	Nasal swab (%)	Nasal wipe (%)	Oropharyngeal swab (%)	Oral fluid (%)	Surface wipe (%)	Udder wipe (%)	Airborne particle deposition (%)	Air (%)
Sow herd	1	POS	8/10* (80)	7/10 (70)	10/10 (100)	3/3 (100)	9/10 (90)	10/10 (100)	10/10 (100)	10/10 (100)
	2	POS	7/10 (70)	6/10 (60)	9/10 (90)	1/1 (100)	7/10 (70)	9/10 (90)	10/10 (100)	10/10 (100)
	3	NEG	0/10 (0)	0/10 (0)	0/10 (0)	0/3 (0)	0/10 (0)	0/10 (0)	0/10 (0)	0/10 (0)
	4	POS	2/10 (20)	3/10 (30)	4/10 (40)	1/2 (50)	3/10 (30)	5/10 (50)	5/10 (50)	10/10 (100)
	5	POS	5/10 (50)	5/10 (50)	8/10 (80)	1/1 (100)	5/10 (50)	7/10 (70)	10/10 (100)	10/10 (100)
	6	NEG	0/10 (0)	0/10 (0)	0/10 (0)	0/1 (0)	0/10 (0)	0/10 (0)	0/10 (0)	0/10 (0)
	Total^&^	–	22/40 (55)^a^	21/40 (53)^a^	31/40 (78)^a^	6/7 (86)^x^	24/40 (60)^x^	31/40 (78)^x^	35/40 (88)^z^	40/40 (100)^z^
Wean-to-finish	1	NEG	0/10 (0)	0/10 (0)	NC**	0/10 (0)	0/10 (0)	NC	0/10 (0)	0/10 (0)
	2	POS	6/10 (60)	6/10 (60)	NC	4/10 (40)	3/10 (30)	NC	0/10 (0)	1/10 (10)
	3	POS	2/10 (20)	2/10 (20)	NC	10/10 (100)	5/10 (50)	NC	9/10 (90)	10/10 (100)
	4	POS	4/10 (40)	4/10 (40)	NC	5/10 (50)	5/10 (50)	NC	7/10 (70)	6/7 (85.7)
	5	POS	2/10 (20)	3/10 (30)	NC	7/8 (87.5)	10/10 (100)	NC	9/10 (90)	10/10 (100)
	6	POS	1/10 (10)	1/10 (10)	NC	4/10 (40)	2/10 (20)	NC	4/10 (40)	4/10 (40)
	Total^&^	–	15/50 (30)^a^	16/50 (32)^a^	NC	30/48(62.5)^x^	25/50 (50)^x^	NC	29/50 (58)^z^	31/47 (65.9)^z^

*Number of rRT-PCR positive/number tested

**NC: Not collected

^&^Total values include only the samples of the influenza positive farms

Superscripts (letters a, x, z) show statistical differences between sample types at each level

Nasal swabs, nasal wipes and oropharyngeal swabs were tested in pools of three

Alpha 0.05 for statistical significance

In wean-to-finish pigs, from the IAV positive herds, 30% (15/50, 95% CI 17–44%) of pooled nasal swabs and 32% (16/50, 95% CI 20–47%) of pooled nasal wipes tested rRT-PCR positive (*p* = 1). Higher positivity rates among the group-level samples were observed with 50% (25/50, 95% CI 36–64%) and 62.5% (30/48, 95% CI 47–76%) oral fluids and surface wipes testing positive, respectively. No statistical difference was observed between oral fluids and surface wipes (*p* = 1). Finally, IAV was detected in 65.9% (31/47, 95% CI 51–79%) of air samples compared with 58% (29/50, 95% CI 43–72%) of airborne particle deposition samples (*p* = 1).

**Fig. 1:**
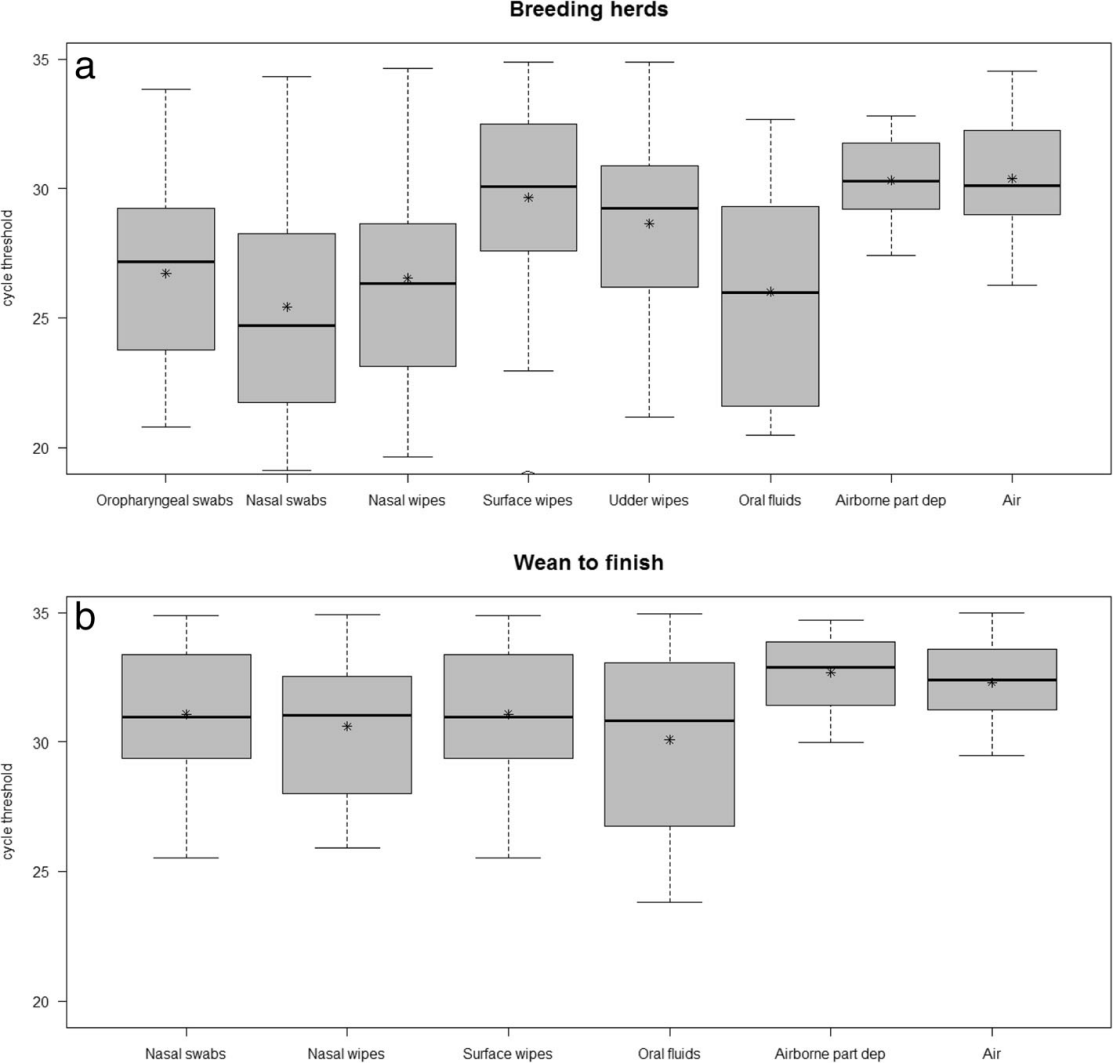
Whisker plots of influenza A virus rRT-PCR cycle threshold (Ct) positive values (Ct ≤ 35) by farm type **a**) breeding herds, **b**) wean-to-finish by sample type

Distribution of rRT-PCR positive Ct values for each sample type and farm type is shown in Fig. 1. Ct values varied depending on the sample type in positive samples collected at both sow and wean-to-finish farms. In general Ct values from sow herd samples were lower than those from wean-to-finish samples. In breeding herds post-hoc tests identified differences (adjusted *p*-value < 0.05) in median Ct values of positive pooled oropharyngeal swabs and nasal swabs compared to other sample types collected at group or room (environmental) level except udder skin wipes (Fig. 1). In wean-to-finish farms, median Ct values for pooled individual and group samples were lower than environmental samples collected at room level, although differences were only found between positive airborne particle deposition and air, oral fluids and surface samples.

### Agreement between tests

Agreement between the different sampling strategies ranged from moderate (Kappa = 0.41–0.60) to substantial (0.61–0.80) in breeding herds (Table 2). In wean-to-finish farms, kappa coefficient showed substantial agreement between pooled nasal swabs and pooled nasal wipes (0.64) and moderate agreement between oral fluids and surface wipes (0.44). Only slight agreement (0.00–0.20) was observed in the case of airborne particle deposition and pooled nasal swabs in wean to finish pigs. Airborne deposited particles and air showed a substantial agreement in breeding herds (0.79) but only moderate agreement in wean-to-finish farms (0.49).

**Table 2:**
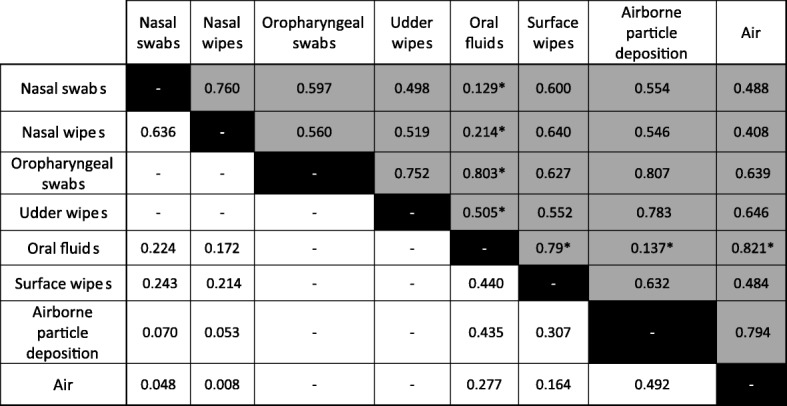
Agreement measured as kappa coefficients (0–1) between sample types collected in breeding herds (right upper side shown in grey) and wean-to-finish farms (left lower site shown in white)

*Coefficient intervals exceeded due to sample size

### IAV isolation

IAV isolation rates varied between farm and sample type (Table 3). In breeding herds, from the rRT-PCR positive samples, IAV was isolated from 78.4% (40/51, 95% CI 65–89%) of oropharyngeal swabs, 62.9% (34/54, 95% CI 49–76%) of nasal swabs, 64.9% (37/57, 95% CI 51–77%) of nasal wipes (*p* > 0.05). IAV was isolated from 47.6% (10/21, 95% CI 26–70%) of surfaces wipes, 75% (18/24, 95% CI 53–90%) of udder wipes, 16.6% (1/6, 95% CI 1–64%) of oral fluids (*p* > 0.05), 29.2% (7/24, 95% CI 13–51%) of airborne deposited particle samples and 34.6% (9/26, 95% CI 17–56%) of air samples (*p* > 0.05). In wean-to-finish farms, IAV was isolated from 47.6% (20/42, 95% CI 32–64%) of nasal swabs, 45% (23/51, 95% CI 31–60%) of nasal wipes (*p* > 0.05), 29.2% (7/24, 95% CI 13–51%) of surface wipes, 5% (1/20, 95% CI 1–25%) of oral fluids (*p* > 0.05), 9.52% (2/21, 95% CI 1–30%) of airborne deposited particle samples and 5.3% (1/19, 95% CI 1–26%) of air samples (*p* > 0.05).

**Table 3:** Isolation of influenza A virus in cell culture by sample and farm type

Farm type	Farm ID	Nasal swabs (%)	Nasal wipes (%)	Oropharyngeal swabs (%)	Oral fluids (%)	Surface wipes (%)	Udder wipes (%)	Airborne particle deposition (%)	Air (%)
Sow herd	1	8/18* (44.4)	10/18 (55.5)	13/18 (72.2)	0/3 (0)	4/9 (44.4)	4/8 (50)	5/9 (55.5)	4/8 (50)
	2	14/15 (93.3)	14/15 (93.3)	15/15 (100)	0/2 (0)	2/5 (40)	7/7 (100)	2/7 (28.5)	4/7 (57.1)
	3	NT**	NT	NT	NT	NT	NT	NT	NT
	4	5/6 (83.3)	6/9 (66.6)	5/6 (83.3)	1/1 (100)	2/2 (100)	2/3 (66.6)	0/3 (0)	1/3 (33.3)
	5	7/15 (46.6)	7/15 (46.6)	7/12 (58.3)	0/0 (0)	2/5 (40)	5/6 (83.3)	0/5 (0)	0/8 (0)
	6	NT	NT	NT	NT	NT	NT	NT	NT
	Total^&^	34/54 (62.9)^a^	37/57 (64.9)^a^	40/51 (78.4)^a^	1/6 (16.6)^x^	10/21 (47.6)^x^	18/24 (75)^x^	7/24 (29.2)^z^	9/26 (34.6)^z^
Wean-to- finish	1	NT	NT	NC***	NT	NT	NC	NT	NT
	2	6/15 (40)	9/18 (50)	NC	0/3 (0)	0/3 (0)	NC	0/1 (0)	1/2 (50)
	3	1/3 (33.3)	2/6 (33.3)	NC	0/6 (0)	0/6 (0)	NC	0/5 (0)	0/4 (0)
	4	8/12 (66.6)	9/15 (60)	NC	0/5 (0)	5/5 (100)	NC	1/7 (14.2)	0/6 (0)
	5	2/9 (22.2)	3/12 (25)	NC	0/5 (0)	1/7 (14.2)	NC	0/5 (0)	0/3 (0)
	6	3/3 (100)	NT	NC	1/1 (100)	1/3 (33.3)	NC	1/3 (33.3)	0/4 (0)
	Total	20/42 (47.6)^a^	23/51 (45)^a^	NC	1/20 (5)^x^	7/24 (29.2)^x^	NC	2/21 (9.5)^z^	1/19 (5.3)^z^

*Number of positive/number tested

**NT: Not tested (these samples were not tested because farm was confirmed influenza negative by rRT-PCR)

***NC: Not collected

^&^Total values include only the samples of the influenza positive farms

Superscripts (letters a, x, z) indicate statistical differences between sample types at each level

Alpha 0.05 for statistical significance

### Sequencing of IAV

Table 4 shows the sequencing success rates for hemagglutinin (HA) and neuraminidase (NA) IAV gene segments stratified by sample type for breeding herds and wean-to-finish farms. The overall number of analyzable sequences (> 900 bp for hemagglutinin and > 800 bp for neuraminidase) obtained from sequencing directly from the sample was low and ranged between 0 and 20%. In general, individual sampling strategies had better sequencing success rates than group or environmental samples, and sequencing rates were higher in samples from breeding herds compared to wean-to-finish herds. Obtained sequences with more than 900 bp for hemagglutinin and 800 bp for neuraminidase were used to construct a phylogenetic tree (Additional file 1 Figure S1 and Additional file 2 Figure S2). Hemagglutinin subtype 1 (H1) sequences correspond to 1A.3.3.3/npdm, 1A.3.3.2/Gamma, 1B.2.2.1/Delta 1 and 1A.1.1.1 Alpha clades while hemagglutinin subtype 3 sequences being of human-like cluster A/Victoria/361/2011/H3N2/huSeas/HUMAN/H3. Neuraminidase subtype 2 (N2) sequences appear to be of the N2–2002 lineage while neuraminidase subtype 1 (N1) sequences appear to be of the classical lineage.

**Table 4:** Number of sequences (%) of hemagglutinin and neuraminidase influenza A virus gene segments obtained directly from the samples by sample type, type of farm and sequence length

Sample type	BREEDING HERDS				WEAN-TO-FINISH HERDS			
	Hemagglutinin		Neuraminidase		Hemagglutinin		Neuraminidase	
	< 900 bp^b^	> 900 bp	< 800 bp	> 800 bp	< 900 bp	> 900 bp	< 800 bp	> 800 bp
Nasal swab	2/54 (3.7)^a^	9/54 (16.6)	0/54 (0)	11/54 (20.4)	2/42 (4.8)	5/42 (11.9)	3/42 (7.1)	4/42 (9.5)
Nasal wipe	1/57 (1.8)	12/57 (21)	0/57 (0)	11/57 (19.3)	1/51 (1.9)	5/51 (9.8)	1/51 (1.9)	4/51 (7.8)
Oropharyngeal swab	0/51 (0)	7/51 (13.7)	2/51 (3.9)	7/51 (13.7)	NC^c^	NC	NC	NC
Oral fluid	0/6 (0)	0/6 (0)	1/6 (16.7)	0/6 (0)	0/20 (0)	0/20 (0)	0/20 (0)	0/20 (0)
Surface wipe	1/21(4.8)	2/21(9.5)	0/20(0)	0/20 (0)	0/24 (0)	1/24 (4.2)	0/24 (0)	0/24 (0)
Udder wipe	1/24 (4.1)	3/24 (12.5)	0/24 (0)	2/24 (8.3)	NC	NC	NC	NC
Airborne particle deposition	2/24 (8.3)	3/24 (12.5)	1/22 (4.5)	1/22 (4.5)	0/21 (0)	0/21 (0)	0/20 (0)	0/20 (0)
Air	0/26 (0)	0/26 (0)	0/26 (0)	0/26 (0)	0/19 (0)	0/19 (0)	0/19 (0)	0/19 (0)
Total	7/263 (2.7)	36/263 (13.7)	4/260 (1.5)	32/260 (12.3)	3/177 (1.7)	11/177 (6.2)	4/177 (2.3)	8/177 (4.5)

^a^Number of sequences/number of total sequencing attempts (percentage)

^b^bp: base pair

^c^NC: Not collected

### Multivariable analysis

In order to evaluate the association between sample type and probability of testing positive by rRT-PCR the final model included the random effects farm and litter/pen, and sample type as a fixed effect. A higher probability of finding a positive result was identified for pooled oropharyngeal swabs, oral fluids, surface wipes and udder wipes compared to pooled nasal swabs (baseline category), while no differences were observed between pooled nasal swabs and pooled nasal wipes. Table 5 shows point estimates and standard errors (SE) of the regression coefficients (β), point estimates and 95% confidence intervals (95% CI) of the adjusted odds ratios (OR) and *p*-values of the association between positive results on samples collected in positive IAV swine farms using a rRT-PCR with a cut-off of 37.5. Area under the receiver operating characteristic (ROC) curve was 0.96 (95% confidence interval 0.945–0.983), suggesting an adequate predictive ability.

**Table 5:** Odds ratios (OR) of finding a positive result by sample type using a multivariate analysis considering two random effects (farm and litter or pen) and using the pooled nasal swab as baseline

Sample type	N	Positive samples / Total samples (%)	β^a^	SE β^b^	OR (95% CI)	*P* value
Nasal swab	90	49/90 (54.4)	0.371	0.702	Reference	–
Nasal wipe	90	51/90 (56.7)	0.199	0.447	1.22 (0.51–2.95)	0.66
Oropharyngeal swab	40	32/ 40 (80)	2.088	0.714	8.07 (2.13–35.7)	0.003
Oral fluid	55	41/55 (74.5)	2.284	0.635	9.81 (3.02–37.3)	< 0.001
Surface wipe	90	62/ 90 (68.9)	1.354	0.478	3.87 (1.55–10.2)	0.004
Udder wipe	40	34/40 (85)	2.803	0.79	16.5 (3.88–89.0)	< 0.001

^a^β: regression coefficient

^b^SE β: Standard error of the regression coefficient

Nasal swabs, nasal wipes and oropharyngeal swabs were tested in pools of three

## Discussion

Surveillance is a key component of influenza prevention, control and elimination plans for pig producers. Samples collected at the group level or from the environment where the pigs are raised can be a suitable option to reduce sample size and facilitate sample collection as long as the sampling provides a high sensitivity for virus detection, good virus isolation rates and subsequent genetic characterization of the viruses sequenced from the samples. Our results indicate that collection of samples at the litter or pen level and environmental samples collected at the room level (i.e air, surfaces) yield better detection rates than samples collected at the individual level from nasal swabs tested in pools. However, individual samples in particular oropharyngeal swabs in suckling pigs and nasal swabs and nasal wipes in growing pigs, and udder wipes in lactating sows were the best samples to obtain an isolate.

Traditionally nasal swabs have been considered the reference sample for detecting and isolating influenza in pigs [12]. More recently oral fluids became the sample of choice for detecting IAV in large populations because of its higher sensitivity at the herd level and ease of collection by non-specialized personnel [13]. However, the ability to recover a viable isolate using this sample type is low. In our study we compared eight different sample types involving the collection of over 1,300 samples from breeding and wean-to-finish farms, and found that the oropharyngeal swab and the udder wipe were the optimum sample type for both detecting and isolating IAV from suckling pigs. Oropharyngeal swabs were not collected in growing pigs because of the difficulty to reach the oropharyngeal cavity, and therefore further research is necessary to determine whether this type of sample is recommended for IAV sampling in growing pigs. In wean-to-finish pigs, isolation rates were higher in nasal swabs than nasal wipes although direct comparison of the test results within each category (individual, group or environmental) between sample types did not reveal differences (*p* > 0.05). This may be of interest since wiping the nose of pigs may be easier and less laborious than collecting nasal swabs.

In breeding herds, we have evaluated for the first time the usefulness of sampling the udder skin of lactating sows to detect IAV, a sample that represents groups of suckling pigs and that resulted in a method capable of identifying positive litters and yielding viable IAV. Detection and isolation rates of IAV from udder wipes were as high as those obtained from oropharyngeal swabs. To the knowledge of the authors detection and isolation of IAV from skin of pigs has not been reported before and we speculate that contact with contaminated skin of lactating sows may be a route of influenza transmission among pigs prior to weaning. Interestingly, in humans IAV has been detected and isolated from skin of infected people and direct contact of contaminated hands has been implicated in the transmission of IAV [14]. Even though there is evidence that milk and mammary gland itself could be IAV infected [15], the most likely source of the virus in the udder skin is the nasal and oral secretions left by pigs during suckling. Pigs suckle frequently, every hour approximately [16] and the pig behaviour of rooting to stimulate milk release appears to favor the contamination of the sow skin with nasal and oral secretions from the piglets during suckling. Our results indicate that sampling the udder skin can be an effective, simple and a practical approach to identify influenza infected litters. The labor for handling pigs to collect individual samples such as oropharyngeal swabs, nasal wipes or nasal swabs, the high sample size required when using individual samples to achieve an adequate sensitivity, in addition to the limitation for collection of oral fluids in litters suggest that udder wipes can be a cost effective and easy option to detect and isolate IAV effectively in pigs prior to wean. Pigs prior to wean are a known reservoir of influenza in breeding herds and a target population for surveillance [17, 18]. In addition, our results suggest that contaminated skin may serve as a means of IAV transmission within and between litters. Further research is needed to evaluate whether contaminated skin of sows can act as fomite in the transmission of IAV and other pathogens. In the same way, further research is also needed to determine if the pig mammary gland and milk therein is infected with IAV as it has been shown for ferrets and humans [15].

Use of oral fluids collected from litters of pigs yielded a high detection rate of IAV, but isolation rates were low and pigs were reluctant to chew on the ropes as reported in other studies [19], which resulted in a low number of samples. In wean-to-finish pigs, eventhough oral fluids were easily collected and IAV detection was good, virus isolation was still poor which is in agreement with previous results [20]. In contrast, surface wipes yielded a lower detection rate but better isolation and sequencing rates than oral fluids which indicates that environmental sampling could be considered as an alternative approach to diagnose IAV in growing pigs.

Overall, sampling the environment appeared to be a good approach to detect IAV since the virus was readily detected from air and samples representing the deposition of airborne particles, although environmental samples yielded fewer isolates than individual or group sample types. Detection of IAV from the environment has implications in the potential transmission and persistence of IAV in pigs and risk of infection to people as reported by Choi et al. [10].

In this study, rRT-PCR positive Ct values were lower and detection rates were higher in suckling pigs in breeding herds compared to wean-to-finish suggesting that there was a higher viral load in pigs prior to weaning than in older pigs. Differences in IAV infection status between farms could explain this difference but nevertheless, pigs of weaning age (e.g. approximately 17–21 days of age) are a recommended target population to determine status, conduct influenza surveillance or obtain viral isolates more efectively. This information is relevant particularly in influenza positive farms in order to make decisions regarding influenza control programs.

The multivariable analysis that compared the performance between sample types while correcting for the lack of independence between samples suggested that in fact there was a better probability of detecting influenza using group samples rather than individual pig samples when comparing it to the standard nasal swab sample. This multivariate analysis supports previous studies in which group samples had a better detection capacity compared with individual pig samples [21, 8]. These results suggest the use of group rather than individual strategies for influenza surveillance in farms when the objective is to determine the infection status of the population.

Sequencing is necessary to genetically characterize IAV strains, stablish genetic diversity and has become routine in surveillance programs. We were interested in sequencing IAV directly from the sample since this is done in diagnostic laboratories when a viral isolate is not available. Overall sequencing rates were higher in individual compared to group and enviromental samples in both, sow and wean-to-finish herds. However, overall sequencing rates were low and our data indicate that sequencing from an isolate is still the method of choice when using Sanger sequencing which is in agreement with previous studies [22] and recommendations from the Center for Disease Control and Prevention [23].

## Conclusions

This study provides new information on sampling approaches to detect, isolate and sequence IAV in swine herds. Our findings suggest that group and environmental sampling strategies are better than individual ones to conduct active surveillance but individual samples may still be needed to obtain a viral isolate or conduct sequencing more effectively. Group samples described in this study are easy to collect, offer high detection rates and overall can be a cost-effective approach for influenza surveillance programs that require an isolate for sequencing. We report for the first time the use of udder skin wipes collected from lactating sows as a novel and sensitive sample type to detect and isolate IAV from litters prior to weaning. In addition, our study highlights the role of the environment and the lactating sow as important sources of IAV in pigs. This information is relevant for influenza control programs directed at minimizing IAV transmission between pigs, from pigs to other species and from pigs to people.

## Methods

### Ethics statement

Protocols and procedures followed during the study were approved by the University of Minnesota Institutional Animal Care and Use Committee (protocol number 1510-33054A) and the Institutional Biosafety Committee (protocol number 1508-32918H). The animals were owned by producers who had provided written consent to have samples collected from the animals.

### Farm and sample selection

A longitudinal prospective study was designed to compare eight distinct sampling procedures to detect, isolate and sequence IAV. Sampling was conducted in pigs prior to wean at approximately 18–21 days of age from breeding herds (*n* = 6), and in weaned growing pigs between 35 and 45 days of age housed in wean-to-finish facilities (*n* = 6).

Herds were located in Minnesota, Iowa and North Dakota and were selected based on their prior history of IAV infection upon consultation with the herd veterinarian. Three pigs from 10 litters distributed in 3 farrowing rooms, or 3 pigs from 10 pens distributed in a single room were selected from each sow herd or wean-to- finish facility, respectively. In order to maximize the probability of detecting IAV-positive samples, pens or litters were selected whenever possible based on presence of pigs with clinical signs of coughing or sneezing suggestive of IAV infection, such as coughing or sneezing, and randomly when clinical signs were not observed.

Samples collected at the individual animal level included nasal swabs, oropharyngeal swabs and nasal wipes. Group samples included oral fluids, wipes from the udder skin of the lactating sows and samples from surfaces in contact with pigs (i.e feeders, pen railing, drinkers). Environmental samples included air and the deposition of airborne particles that had settled by gravity on surfaces.

For the individual samples, sample size was set to detect a within-farm IAV prevalence of 15% assuming a 90% sensitivity, 100% specificity and a level of confidence of 95%. Ten pools of 3 samples each (30 samples per farm) were required. Sample size for environmental and group samples was set to detect a prevalence of 30% based on prior work by Neira et al. [11] using the same sensitivity, specificity and confidence level, which led to a sample size of 7 that was rounded up to 10 samples per farm. All sample size calculations were carried out using the Epitools epidemiological calculator [24].

### Sampling procedures

Nasal and oropharyngeal swabs were collected from individual pigs using rayon-tipped swab applicators with Stuart’s medium (BBL CultureSwab™ liquid, Stuart single plastic applicator; Becton, Dickinson and Com. Sparks, MD, USA). Nasal swabs were collected by inserting the swab approximately 2–4 cm into each nostril and rotating the swab gently. Oropharyngeal swabs were collected by manually opening the mouth of the pig and inserting the swab towards the caudal part of the mouth and rotating the swab along the sides of the mouth and its deeper portion (pharynx). Oropharyngeal swabs were not collected in growing pigs due to the difficulty to reach the oropharyngeal cavity with the methods employed. Nasal wipes were collected by wiping the exterior of the snout of the pigs. Surface samples from feeders, drinkers and pen railings were collected by thoroughly wiping surfaces in contact with the mouth and noses of the pigs. The surface of the udder skin was wiped thoroughly in the areas of contact between the pig’s nose and udder skin during or after suckling in order to collect the secretions left by the litter during suckling. In order to measure the deposition of airborne particles on surfaces, 1 m by 0.3 m (0.3m^2^) of foil paper was placed flat on top of a crate or feeder. The foil paper was left untouched for 60 min and then the surface was wiped with a gauze.

All surfaces were sampled using wipes which were prepared with a 3 × 3 in. sterile gauze impregnated with 10 ml of DMEM-Dulbecco’s Modified Eagle Medium Gibco™ (Grand Island, NY, USA) supplemented with antibiotics and antimycotics. The gauzes were individually bagged and kept frozen at − 20 °C until use. During sampling, surfaces were wiped thoroughly and after that, each gauze was placed back in its individual bag, and transported to the laboratory for testing using ice packs to keep the samples refrigerated.

Oral fluids were collected using a cotton rope (WebRiggingSupply.com, Barrington, IL, USA) as previously described by Prickett et al. [21]. Briefly, a 0.5 m rope was placed hanging either from a farrowing crate or a pen for pigs to chew on it. After approximately 30 min, ropes were squeezed into a Ziploc bag and fluid collected in a disposable plastic tube and transported to the laboratory for testing using ice packs to keep the samples refrigerated.

Air samples were collected using an air cyclonic sampler (Midwest Micro-Tek, Brookings, SD, USA) capable of sampling 200 L of air per min as previously described [11]. Briefly, 10 mls of DMEM were added to the air sampler collection vessel and sampler operated for 30 min. After collection, the liquid in the collection vessel was transferred into a tube and transported to the laboratory. Gloves were changed between the collection of each sample to avoid cross contamination. Finally, air samples were placed in ice packs immediately after collection and transported to the laboratory for processing and testing.

### Diagnostic tests

All samples were processed for viral RNA extraction using the magnetic particle processor procedure (Ambion® MagMAX™AM1835, Viral RNA Isolation Kit; Applied Biosystems, Foster City, CA, USA) and tested by real time RT-PCR (rRT-PCR) to detect the IAV matrix gene [25]. Nasal swabs, nasal wipes and oropharyngeal swabs were tested in pools of three within litters, while the rest of samples were tested individually. Results with cycle threshold (ct) value ≤35 were considered positive, ct > 35 and ≤ 40 suspect and ct > 40 negative.

A selected number of rRT-PCR positive samples from each sample type were cultured for virus isolation using Madin-Darby canine kidney (MDCK) cells [20, 26]. All samples were tested individually. MDCK cells were prepared in 6-wells plates for each selected sample. Wells were inoculated with 200 μl and 100 μl of sample, in duplicate, and incubated for 1 h at 37 °C with 5% CO_2._ 1.5 ml of DMEM media (Gibco™, Grand Island, NY, USA) supplemented with 7.5% bovine serum albumin (Gibco™, Grand Island, NY, USA), 1X antibiotics and antimycotic (Gibco™, Grand Island, NY, USA), 750 μl 1 mg/ml trypsin-TPCK, gentamicin, neomycin was added to each well, then the plates were incubated at 37 °C with 5% CO_2_. Plates were evaluated at day 3 and 5 for appearance of positive cytopathic effect (CPE). All the wells with positive CPE were confirmed by hemagglutination assay (HA) using 0.5% turkey red blood cells and VetScan Avian Influenza Type A Virus Rapid Test (Alere Scarborough Inc., Union city, CA, USA).

Hemagglutinin (HA) and neuraminidase (NA) IAV gene segments were targeted for sequencing. Viral RNA was obtained directly from the samples selected for virus isolation with a QIAamp RNeasy Mini Kit (QIAGEN, Inc., Valencia, CA), using the protocol recommended by the manufacturer. rRT-PCR amplification of each gene segment was performed under standard conditions [25]. Sequencing primers were used based on a previous report [27]. For HA, rRT-PCR products were then purified using a QIAamp Gel extraction kit (QIAGEN) and then submitted for Sanger sequencing at the University of Minnesota Genomics Center using a fully automated ABI 3730xl DNA Analyzer (Perkin-Elmer SeqGen, Inc. Torrance, CA, USA) with ABI BigDye Terminator version 3.1 chemistry - Perkin-Elmer (Applied Biosystems 2002). Once sequences were obtained, these were aligned and assembled using Geneious 9.1.7 [28] (http://www.geneious.com). Sequences were finally blasted using the NIAID Influenza Research Database (IRD). Data was obtained from the NIAID Influenza Research Database (IRD) [29] for subtyping and for determining the family clade/cluster classification [30]. In addition, a minimum of 900 nucleotide base pairs (bp) for hemagglutinin and 800 for neuraminidase gene segments were considered the appropriate length useful for analysis [5]. Shorter sequences were noted as partial sequences and not analyzed.

### Statistical analysis

Differences in the results by sample type in each farm type were compared using the Cochran Q test followed by paired McNemar test adjusting the *p*-value for multiple comparisons using the Bonferroni method. Quantitative Ct values found in positive (Cts ≤35 cycles), suspect (Cts > 35 - ≤40 cycles) and negative samples (Cts > 40) from individual, group and environmental samples were compared using paired Wilcoxon signed-rank tests adjusting the p-value for multiple comparisons using the Bonferroni method. All these analyses were carried out in R version 3.3.3 (R Core Team, 2013). The agreement between the qualitative rRT-PCR results was evaluated using the kappa statistic in SPSS 22.0 (IBM, Boulder, CO, USA).

The association between sample type and probability of testing positive by rRT-PCR was then evaluated using a multivariate generalized lineal mixed model in which test result (positive/negative) was the outcome variable and sample type, farm type (breeding/wean-to-finish) and the interaction between the two were considered as candidate fixed effects. In addition, litter/pen and farm were considered as potential random effects to account for the lack of independence between observations obtained from the same pen/litter and/or farm. For this analysis, results were classified as positive or negative using a Ct of 37.5 as the cut-off value as previously reported [31].

The optimal random effects structure was first determined by comparing models that included both potential fixed effects (sample and farm type) and either or both random effects (pen/litter and/or farm) using a likelihood ratio test (LRT) and selecting the one with a lower Akaike’s information criterion (AIC) [32]. Then, significance of the fixed effects was evaluated through the LRT using alpha of 0.05 as the threshold for statistical significance. The predictive performance of the final model was evaluated using receiver operating characteristic (ROC) curves. All analyses were carried in R using the lme4 [33] and pROC [17] packages.

## Additional files

Additional file 1: **Figure S1.** Phylogenetic tree. Phylogenetic tree constructed with obtained sequences from breeding herds. (PDF 153 kb)

Additional file 2: **Figure S2.** Phylogenetic tree. Phylogenetic tree constructed with obtained sequences from weaned growing pigs. (PDF 133 kb)

## Abbreviations

DAP: Deposition of airborne particle
IAV: Influenza A virus
MDCK: Madin-Darby canine kidney
NS: Nasal swabs
NW: Nasal wipes
OF: Oral fluids
OS: Oropharyngeal swabs
RNA: Ribonucleic acid
rRT-PCR: Reverse transcription polymerase chain reaction
SW: Surface wipes
UW: Sow udder skin wipes
