# Supplementary figures and images for: Comparison of individual, group and environmental sampling strategies to conduct influenza surveillance in pigs
Source: BMC Vet Res. 2019 Feb 14;15:61. doi: 10.1186/s12917-019-1805-0 (PMC6376652; doi:10.1186/s12917-019-1805-0)

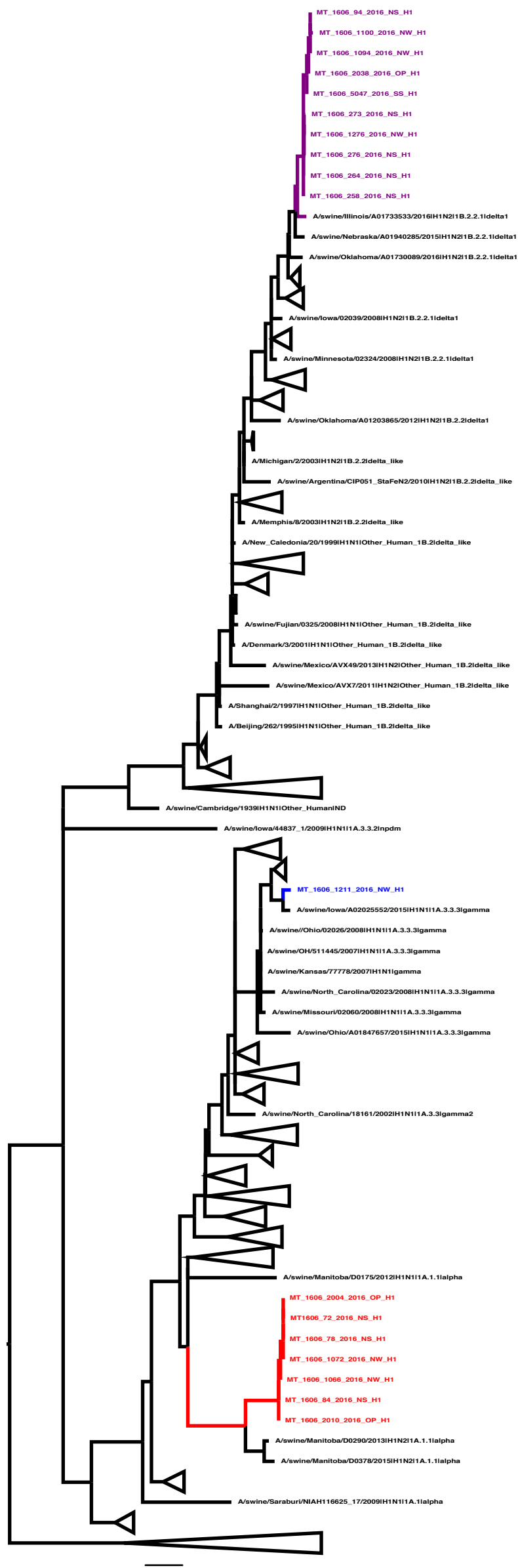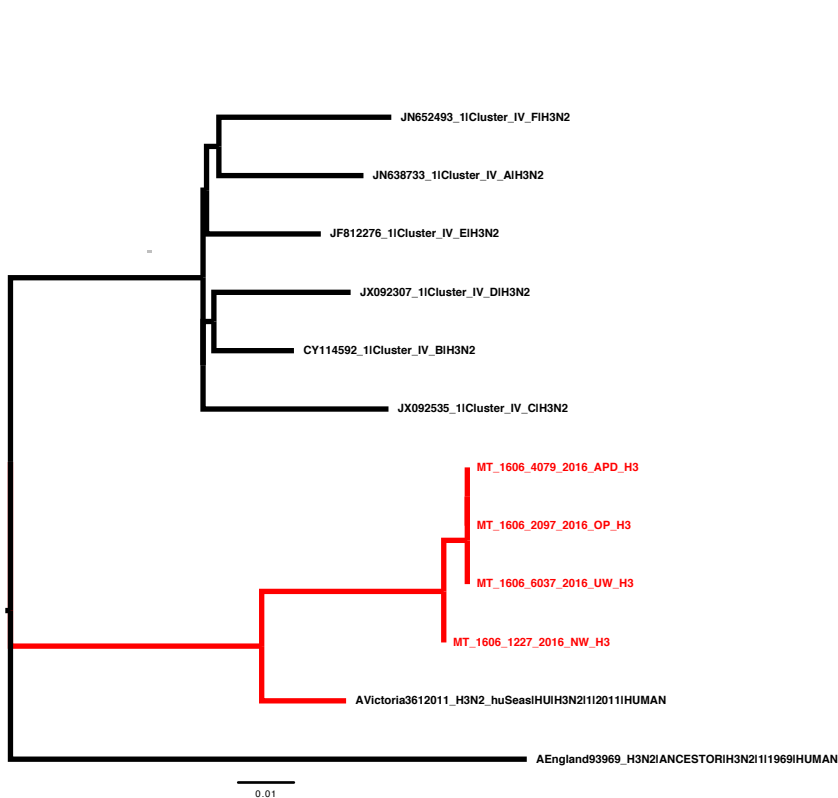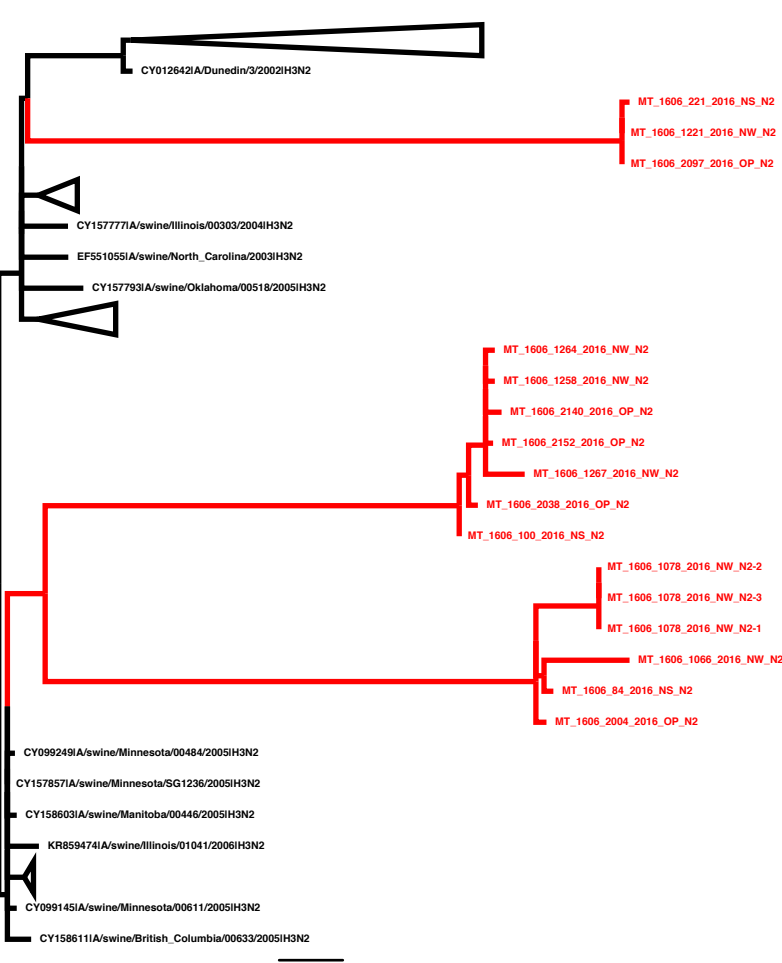

Supplement: Supplementary file 1 — Figure S1. Phylogenetic tree. Phylogenetic tree constructed with obtained sequences from breeding herds. (PDF 153 kb) [file 12917_2019_1805_MOESM1_ESM.pdf]

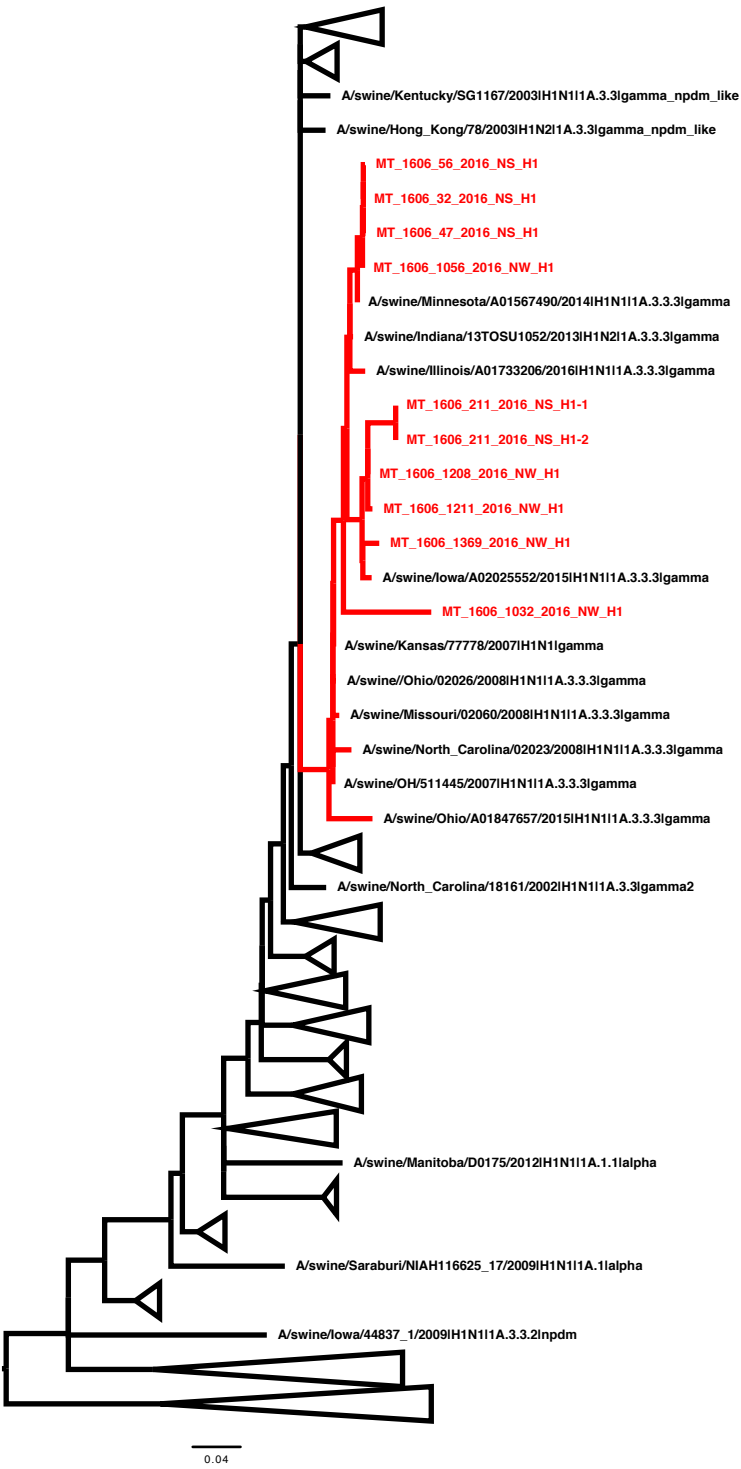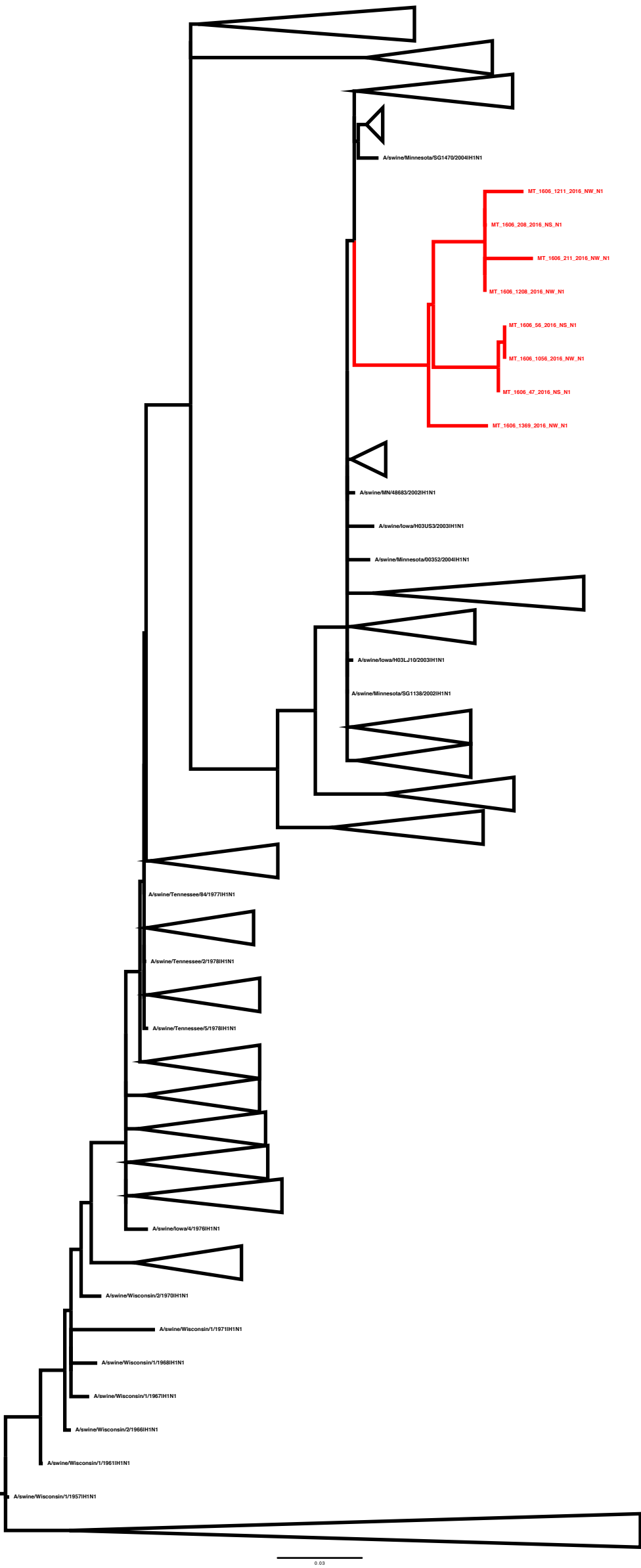

Supplement: Supplementary file 2 — Figure S2. Phylogenetic tree. Phylogenetic tree constructed with obtained sequences from weaned growing pigs. (PDF 133 kb) [file 12917_2019_1805_MOESM2_ESM.pdf]
